# Psychiatric genetics, neurogenetics, and neurodegeneration

**DOI:** 10.3389/fgene.2014.00467

**Published:** 2015-01-14

**Authors:** Berit Kerner

**Affiliations:** Semel Institute for Neuroscience and Human Behavior, University of California, Los AngelesLos Angeles, CA, USA

**Keywords:** risk gene identification, functional genomic variants, social epigenetics, heterogeneity, pleiotropy, amyotrophic lateral sclerosis (ALS), genome-wide association studies (GWAS), environmental exposure

Neuropsychiatric disorders are common, complex, and severe disorders that affect the core of a person: their emotions, intellect, and ability to self-regulate. Millions of individuals worldwide suffer from these disorders; nevertheless, the factors that lead to the manifestation of symptoms are poorly understood. Neuropsychiatric disorders are characterized by heterogeneity, which means that disorders with similar symptoms and disease course do not necessarily share the same disease mechanisms or risk factors. This complex scenario calls for novel and innovative methodological approaches but also for new hypotheses about disease mechanisms and disease classifications. The diverse contributions to this research topic reflect various approaches and opinions in the field of neuropsychiatric genetics and neurodegeneration and could be understood as innovative responses to the challenges in the research on common complex disorders. The authors of this special research topic call for the consideration of genetic and environmental risk factors, and a close attention to the phenotype. The new focus on rare functional genomic variants signals recent changes in the perception of common complex disorders as being not vastly different from rare Mendelian disorders. This shift in perception calls for a reevaluation of common experimental and statistical approaches and the exploration and development of novel and less well-established methods.

The five publications summarized under this research topic address the paradigm shift in common complex disorders in unique ways. Using personality traits in centennials as an example, Bae et al. demonstrate and discuss the analytical and translational challenges of Genome Wide Association Studies (GWAS) in common complex disorders brought about by population-specific allele frequencies, which often hinder the replication of statistically significant results (Bae et al., 2013). Rogaev highlights the importance of rare and highly penetrant mutations in gene-coding regions in familial forms of common complex disorders and draws attention to the limitations of GWAS in these scenarios, using Alzheimer's disease as an example (Rogaev, 2012). He also emphasizes the unparalleled opportunities provided by next-generation sequencing techniques in the search for *de novo* mutations in family trios. However, the interpretation of the vast amount of rare and *de novo* mutations with unknown functional consequences, remains challenging. Krebs et al. uses the example of movement disorders to demonstrate the genetic pleiotropy and heterogeneity governing the relationship between genotype and phenotype (Krebs and Paisán-Ruiz, 2012). Pleiotropy refers to the observation that variations in the same gene can influence several seemingly unrelated phenotypic traits. Heterogeneity describes the observation that the same disease can be caused by genetic variation in any one of a number of genes. The authors also highlight recent successes in risk gene identification that were brought about with the help of next-generation sequencing in combination with linkage studies and/or functional studies in small animals and cell cultures. Sequeira et al. explore the contributions of brain-region specific somatic mutations in mitochondrial DNA in severe psychiatric disorders (Sequeira et al., 2012). And finally, Brigati et al. present the field of social epigenetics, which explores how the wiring of our brain is continuously shaped in response to a constantly changing environment (Brigati et al., 2012). The researchers contemplate how musical experience could shape and be vividly retained within our brain, and how it could affect our behavior.

The multitude of potential disease mechanisms in neuropsychiatric disorders undeniably raises the need for a broad spectrum of analytical approaches. Therefore, it is encouraging to see the variety of hypotheses, study designs, and methods in the field of neuropsychology, as demonstrated in the diverse contributions to our research topic. While over the past decade the focus has been on GWAS to detect common genomic variants as risk factors for neuropsychiatric disorders, the research community is moving its attention to environmental risk factors and gene-environment interactions [European Network of National Networks studying Gene-Environment Interactions in Schizophrenia (EU-GEI) et al., 2014; Wang et al., 2014]. However, funding priorities at the NIH are only slowly following the trend. Taking schizophrenia as an example, the percentage of funded projects that focus on genetic risk factors was 35% in 2011, but only 4% of the funded proposals studied environmental risk factors, and 0.8% focused on epigenetics. In 2014, the percentage of funded genetic studies has declined to 27%, but the percentage of studies with environmental focus also has dropped (to 3%). Funded epigenetic studies dropped to 0.6% of the portfolio. Despite limited contributions to our understanding of disease pathomechanisms, grants using GWAS to identify common genomic risk factors still dominate and constitute a large portion of the research portfolio at NIH.

The success of a more diversified genetic approach to complex phenotypes, including GWAS, linkage analysis and positional cloning, candidate gene studies, exome sequencing, and genome sequencing, has already been demonstrated in, for example, two highly co-morbid disorders: amyotrophic lateral sclerosis (ALS) and frontotemporal lobar degeneration (FTLD; Janssens and Van Broeckhoven, 2013; Marangi and Traynor, 2014). ALS is a rare, heterogeneous, complex neurodegenerative disorder, which affects mostly individuals 65 years of age and older. Genetic risk factors are believed to explain two-thirds of familial cases and about 10% of sporadic cases. Environmental risk factors have also been proposed. So far, a variety of potential genetic disease mechanisms have been proclaimed, including expansion repeats, common and rare inherited genetic variations, de novo mutations, epigenetic changes, somatic mutations, and epistasis. FTLD is also a relatively rare, complex, neurodegenerative disorder. Recent evidence supports the hypothesis that ALS and FTLD are distinct disorders with unique pathomechanisms and overlapping phenotypes. In both disorders, the fused-in-sarcoma (FUS) protein accumulates in the cytosol of neuronal cells in a subset of cases, but in FTLD the protein is methylated, whereas in ALS it is not (Dormann et al., 2012). This modification affects the nuclear import of the protein. A focused approach on familial cases with autosomal dominant inheritance, and the use of FUS protein accumulations as a specific neuropathologic endophenotype present in a subset of cases, led to the identification of disease-causing rare mutations in the FUS gene. These mutations account for about 4% of familial ALS cases (10%). Disease-causing mutations in sporadic ALS cases (90%) and in FTLD remain to be discovered (Da Cruz and Cleveland, 2011). It remains to be seen if similar success can also be accomplished in common, complex psychiatric disorders, in which GWASs in increasingly larger population samples have so far dominated the research portfolio but the identification of disease causing mutations and the discovery of pathomechanisms remain elusive.

Hopefully, funding agencies will follow the common trend toward diversity and encourage novel and creative approaches to risk factors in neuropsychiatric and neurodegenerative disorders. In some of the underdeveloped fields of research, standardized approaches and methods will still need to be developed. In this dynamic process, adherence to solid reporting practices and independent replication of promising results will be essential. However, without funding, innovative ideas will remain what they are—just ideas.

## Conflict of interest statement

The author declares that the research was conducted in the absence of any commercial or financial relationships that could be construed as a potential conflict of interest.
